# CSF biomarkers for early-onset Alzheimer's disease in Chinese population from PUMCH dementia cohort

**DOI:** 10.3389/fneur.2022.1030019

**Published:** 2023-01-09

**Authors:** Dan Lei, Chenhui Mao, Jie Li, Xinying Huang, Longze Sha, Caiyan Liu, Liling Dong, Qi Xu, Jing Gao

**Affiliations:** ^1^State Key Laboratory of Complex Severe and Rare Diseases, Department of Neurology, Peking Union Medical College Hospital, Chinese Academy of Medical Science and Peking Union Medical College, Beijing, China; ^2^State Key Laboratory of Medical Molecular Biology, Institute of Basic Medical Sciences and Neuroscience Center, Chinese Academy of Medical Sciences and Peking Union Medical College, Beijing, China

**Keywords:** early-onset, Alzheimer's disease, CSF biomarkers, cutoff value, combination

## Abstract

**Introduction:**

Alzheimer's disease (AD) is one of the highly concerned degenerative disorders in recent decades. Though vast amount of researches has been done in various aspects, early-onset subtype, however, needs more investigation in diagnosis for its atypical manifestations and progression process. Fundamental CSF biomarkers of early-onset AD are explored in PUMCH dementia cohort to depict its laboratory characteristics.

**Materials and methods:**

A total of 125 individuals (age of onset <65 years old) from PUMCH dementia cohort were recruited consecutively and classified into AD, non-AD dementia, and control groups. Levels of amyloid-β 42 (Aβ42), total tau (t-tau) and phosphorylated tau (p-tau) were measured using ELISA INNOTEST (Fujirebio, Ghent, Belgium). Students' *t*-test or non-parametric test are used to evaluate the differences between groups. Area under curve (AUC) of receiver operating characteristic (ROC) curve was introduced to prove the diagnostic powers of corresponding markers. Logistic regression is used to establish diagnostic model to combine several markers together to promote the diagnostic power.

**Results:**

The average of all three biomarkers and two calculated ratios (t-tau/Aβ42, p-tau/Aβ42) were statistically different in the AD group compared with the other two groups (*Ps* < 0.01). From our data, we were able to provide cutoff values (Aβ42 < 570.9 pg/mL; p-tau > 56.49 pg/mL; t-tau > 241.6 pg/mL; t-tau/Aβ42 > 0.529; p-tau/Aβ42 > 0.0846) with acceptable diagnostic accuracy compared to other studies. Using a combination of biomarkers and logistic regression (area under curve 0.951), we were able to further improve diagnostic efficacy.

**Discussion:**

Our study supports the diagnostic usefulness of biomarkers and defined cutoff values to diagnose early-onset AD. We showed that the ratios of t-tau/Aβ42 and p-tau/Aβ42 are more sensitive than relying on Aβ42 levels alone, and that we can further improve diagnostic accuracy by combining biomarkers.

## 1. Introduction

Early diagnosis of Alzheimer's disease (AD) helps patients receive the treatment and support they need. Screening for amyloid-β 42 (Aβ42), total-tau (t-tau), and phosphorylated-tau (p-tau) in CSF has shown promise in detecting AD (1). Combined with official criteria for diagnosing AD, CSF profiles improve diagnostic accuracy (2–8). However, high variability within and between different populations impedes development of an international standard (9). In particular, China possesses over 1/5 of the world's population, and over 10 million AD patients, but few well-accepted studies have looked at these biomarkers of early-onset AD sub-group. Therefore, we conducted our own study to explore characteristics and estimate cutoff values for these biomarkers specific to patients under the age of 65, the early-onset sub-type of AD.

Early-onset AD differs from late-onset AD in both clinical manifestations and pathological patterns, and its diagnosis is often delayed (10). A substantial percentage of early-onset AD patients have various phenotypes—including logopenic progressive aphasia, frontal lobe variant, cortical basal syndrome, and posterior cortical atrophy (11, 12)—uncharacteristic of typical AD; these patients are frequently misdiagnosed (13). Furthermore, early-onset AD is associated with atrophy in the parietal cortex as opposed to the temporal lobe and hippocampus seen in late-onset AD (14, 15). Nevertheless, both forms of AD show similar CSF profiles: low levels of Aβ42, elevated t-tau and p-tau (16).

As apparently emphasized in the framework of National Institute on Aging and Alzheimer's Association (NIA-AA) in 2018, no universal cut-off value can be applied throughout the world, a reliable cut-off value is urgent for our scientific research and clinical practice. By studying this, we hope to improve early diagnosis for those with atypical symptoms of early-onset AD for our following research and enrich the biomarker database.

## 2. Materials and methods

### 2.1. Standard protocol approvals, registrations and patient consents

All patients gave written informed consent and all procedures were undertaken with the approval of the institutional review board of Peking Union Medical College Hospital (No. ZS2009).

### 2.2. Participants

**Figure 1:**
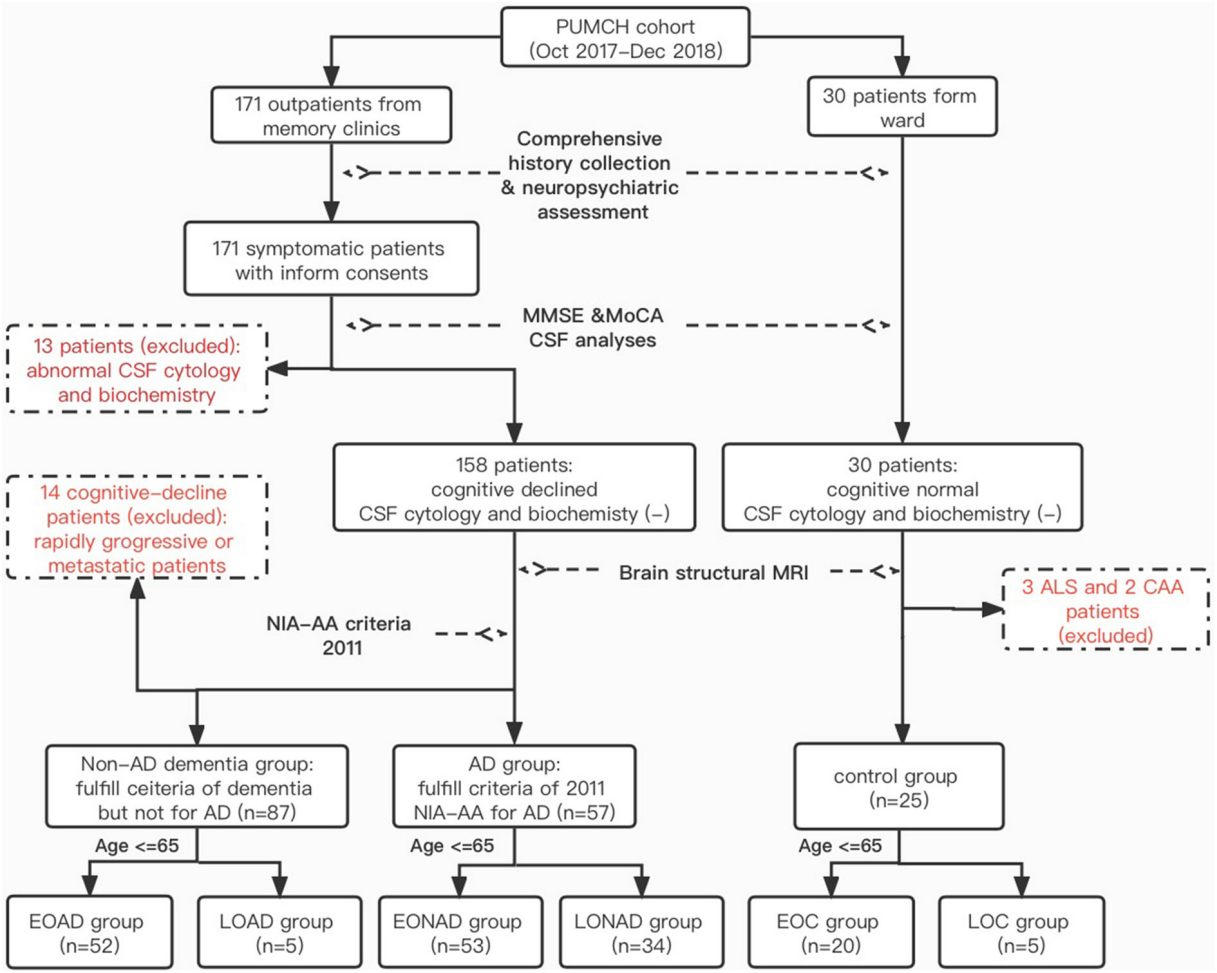
Inclusion and exclusion criteria. *PUMCH, Peking Union Medical Collage Hospital; NIA-AA, National Institute on Aging and Alzheimer's Association; MMSE, Mini-Mental State Examination; MoCA, Montreal Cognitive Assessment; EO(LO)AD, early-onset (late-onset) Alzheimer's disease; EO(LO)NAD, early-onset (later-onset) non-Alzheimer dementia; EO(LO)C, early-onset (late-onset) control; ALS, amyotrophic lateral sclerosis; CAA, cerebral amyloid angiopathy.

A total of 201 patients who visited the Neurological Department at Peking Union Medical College Hospital (PUMCH) in Beijing between October 2017 and December 2018 were enrolled in our PUMCH dementia cohort. Of these, 171 patients had presented to the Memory and Leukoencephalopathy Clinic with cognitive or functional impairment, and 30 patients had no cognitive problems but needed diagnostic lumbar puncture from the ward. The inclusion and exclusion criteria are detailed in Figure 1. Since we were interested in studying biomarkers of early-onset AD, we included only those patients who were younger than 65 when their symptoms manifested (*n* = 139/201; 69.2%). AD (*n* = 52/139; 37.4%) was diagnosed using the 2011 NIA-AA AD criteria (3). Other categories of dementia (*n* = 53; 38.1%) were defined using the Diagnostic and Statistical Manual of Mental Disorders, 5th Edition (DSM-V) (17) (for more details see Table 1). The non-dementia control group (*n* = 20; 14.4%) included patients with Mini-Mental State Examination (MMSE) scores above 26 who showed no cerebral anomaly based on imaging or objective cognitive disturbance on neuropsychological examination. Patients with prion diseases (*n* = 7), cancer metastasis (*n* = 4), or rapidly progressive dementia (*n* = 3) were excluded from this analysis, leaving a sample size of 125 participants.

**Table 1:** Demographic data and biomarker characteristics.

	**AD group**	**Non-AD dementia**	**Control**
Number (M)	52 (20)	53 (28)	20 (10)
Subcategory (*n*)	AD possible (26) AD probable (26)	NPH (9) FTLD (14) Leukoencephalopathy (9) VaD (3) CBD (2) 2° to schizophrenia (2) Other unclarified (14)	MS (4) PN (4) Spinal focal lesion (3) Meningitis (2) Autoimmune (2) Depression (1) Other undiagnosed (6)
Age	57.83 [56.04, 59.61]	53.38^*^ [51.09, 55.66]	47.50^*^ [40.53, 54.47]
MMSE	10.91 [8.73, 13.1]	16.9^*^ [14.4, 19.4]	27.75^†^ [25.04, 30.46]
Aβ42 (pg/mL)	474.3 [441.6, 506.9]	619.2^*^ [558.5, 679.9]	624.6^*^ [512.2, 736.9]
T-Tau (pg/mL)	685.3 [558.6, 812.0]	182.2^†^ [139.4, 224.9]	168.1^†^ [88.52, 255.7]
P-Tau (pg/mL)	86.34 [75.22, 97.45]	39.65^†^ [34.40, 44.91]	34.07^†^ [26.42, 41.73]
T-Tau/Aβ42	1.52 [1.19, 1.85]	0.346^†^ [0.245, 0.447]	1.247^†^ [−0.841, 3.335]
P-tau/Aβ42	0.196 [0.165, 0.227]	0.0766^†^ [0.0618, 0.0915]	0.153^†^ [−0.0525, 0.358]

Data are described as “Mean [95% confidence interval],” except individuals' number “Number (males);” MMSE, Mini-Mental State Examination; NPH, normal pressure hydrocephalus; FTLD, frontotemporal lobe dementia; CBD, corticobasal degeneration; MS, multiple sclerosis; PN, peripheral neuropathy.

* p < 0.01 or

† p < 0.0001, respectively, compared with AD group.

### 2.3. Neuropsychological assessment and CSF sampling

All participants were systematically evaluated by two or more well-trained neurologists for diagnosis and follow-up. Enrolled patients were assessed using the MMSE, Montreal Cognitive Assessment (MoCA), Activities of Daily Living (ADL), Hospital Anxiety and Depression scale (HADs), and systemic cognitive assessments covering at least four functional domains, including but not limited to memory, visuospatial, language, and executive abilities (18). Brain MRI (T1, T2 FLAIR, DWI, ADC, and T2^*^) was routinely assessed. Patients underwent lumbar puncture (LP) within 1 month of their cognitive and MRI assessments (19), with full informed consent. All CSF samples were collected in low protein binding tubes (Eppendorf Protein LoBind Tube, 1.5 mL; Hamburg, Germany) and centrifuged at 1,800 g for 10 min at 4°C. The supernatant was transferred to a new tube and stored at −80°C (20). Commercially-available ELISA kits were used to measure CSF t-tau, p-tau, and Aβ42 [INNOTEST hTAU Ag, PHOSPHO-TAU and β-AMYLOID (1–42); Fujirebio, Ghent, Belgium] in batch within 2 weeks of sampling. All diagnoses were made without any knowledge of CSF protein levels. All individuals were followed-up every 3–6 months until the end of the study.

### 2.4. Internal quality control

Since measurement procedures or different batches of ELISA kits may introduce bias, we routinely ran quality control on our CSF samples. Five randomly chosen samples from our previous work were remeasured using new kits and reagents, directly from the stored low protein binding tubes every 6 months. We also recently remeasured all samples to check for internal bias. No statistically significant differences were found between the new and old biomarker levels (paired *t*-test, *P* > 0.05). Of note, at the beginning of our study, we were using normal tubes to store the CSF samples. However, we later switched to low protein binding tubes. We tested five randomly-selected patients and found no significant difference in the levels detected in the two different types of tubes (Students' *t*-test, *P* > 0.05).

### 2.5. Statistical analysis

In addition to the three original biomarkers, we tested two ratios, t-tau/Aβ42 and p-tau/Aβ42. The normality of the continuous variables was tested using the Shapiro-Wilk test, which showed that the biomarker distributions were not Gaussian in non-AD and control groups. Analyses of the means between any two groups were conducted using non-parametric tests. Receiver operating characteristic (ROC) curves were depicted for every single item, and area under the curve (AUC) was used to describe diagnostic efficacy. As reported in other studies, combining two or more biomarkers can improve diagnostic efficacy (21, 22). Binary logistic regression was used for biomarker combination diagnosis to magnify the accuracy (23). Factor R, constructed from logistic regression, was used for the diagnostic analysis with the other five items. Cutoff values calculated from ROC curves were chosen based on maximum Youden index (YI, sensitivity + specificity-1) (24). Data analyses and figure creation were conducted using SPSS statistics 23.0 (IBM Corporation, Armonk, New York, US) and Graphpad Prism 5.0 (GraphPad Software. Inc, San Diego, California, US). Any difference with *P* < 0.05 was regarded as significant.

## 3. Results

### 3.1. Demographic characteristics and CSF biomarker levels

**Figure 2:**
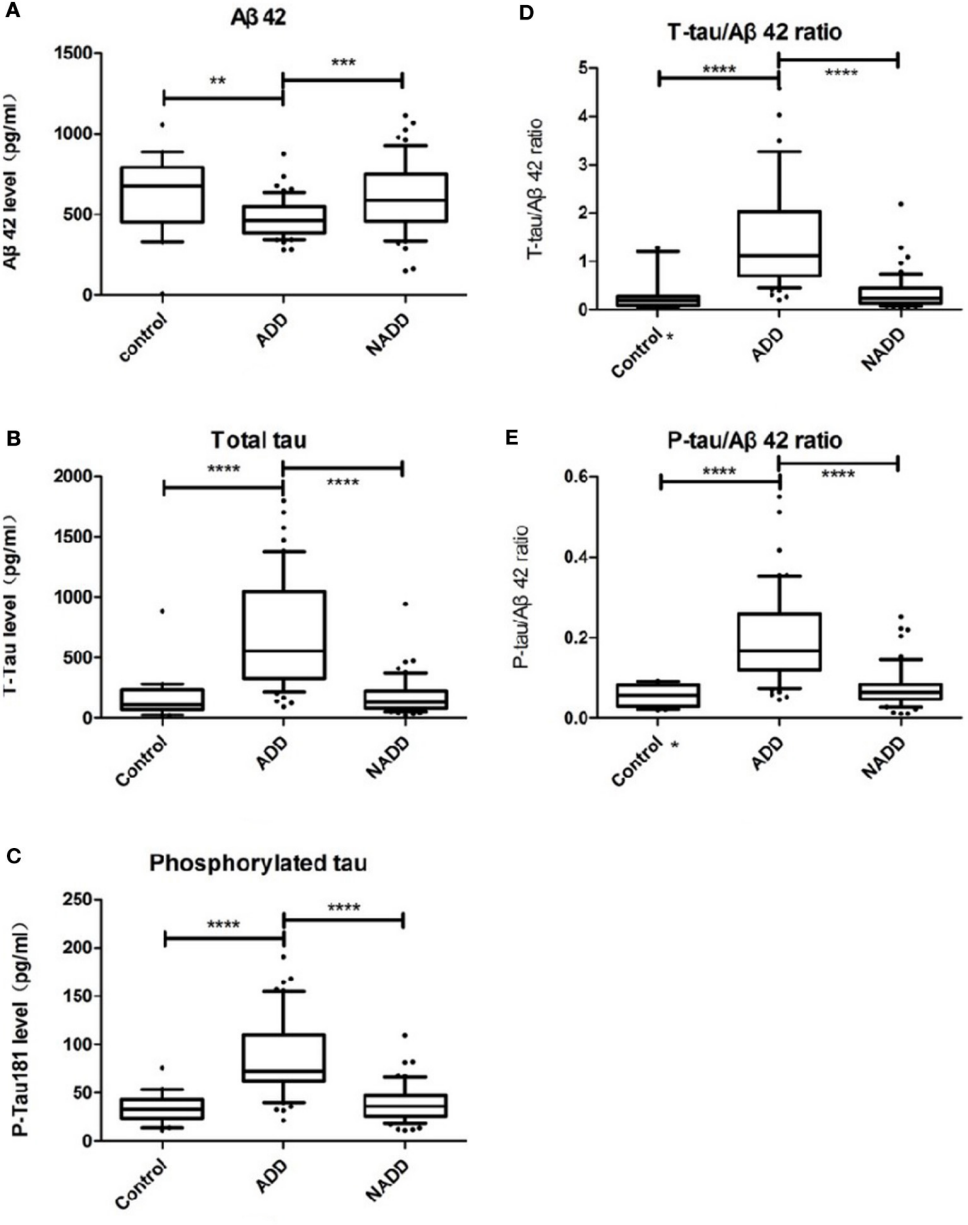
Comparison of CSF biomarker levels across groups. **(A–E)** Display Aβ42, t-tau, p-tau, t-tau/Aβ42, p-tau/Aβ42 distributions of the three groups respectively. Box plots present median and interquartile range and whiskers indicate 10–90% interval. In graph **(D, E)** asterisk markers (*) on X axis indicate that there is an outlier value beyond the upper limit. ADD, AD dementia; NADD, non-AD dementia. **P < 0.01; ***P < 0.001; ****P < 0.0001, between the two groups linked.

Demographic characteristics and CSF biomarker levels of participants are shown in detail in Table 1. We found a significant decrease in Aβ42, and a significant increase in t-tau, p-tau, t-tau/Aβ42 and p-tau/Aβ42, in the AD group compared with both the non-AD and control groups (*Ps* < 0.01, Table 1). Differences were detected in age and MMSE across the different groups (Students' *t*-test, *P* < 0.01). To exclude the potential connection between age, cognitive function, and biomarker levels, correlation analyses were performed. However, we found no relationship between age and biomarker levels, nor MMSE scores and biomarker levels (*Ps* > 0.05, see Table 1). Thus, all five items were considered as reliable and predictable for early-onset AD diagnosis. These differences are displayed graphically in Figure 2.

As reported in previous studies, we found that Aβ42 levels were reduced in AD patients compared with the other two groups, while the levels of the other biomarkers were greatly elevated. All biomarkers other than Aβ42 could be used to differentiate AD with *P* < 0.0001, which supports the diagnostic efficacy later reported in Section 3.3. To identify any outliers, we graphed the results in box plots with the whiskers representing the 10–90% interval (Figure 2). Of note, there was a patient in the control group diagnosed with peripheral neuropathy who had an Aβ42 level of 11.92 pg/mL (confirmed by retest), far lower than the average concentration of 621.3 pg/mL. Because of this outlier Aβ value, the corresponding t-tau/Aβ42 exceeded 20 and p-tau/Aβ42 reached 2.01, both of which are located beyond the upper limits, and thus are not displayed in Figures 2D, E.

### 3.2. Biomarker combination analysis

Since CSF protein levels are continuous variables, relying on a single cutoff value based on statistics is usually insufficient to define a physical or pathological change. We wanted to test whether combining all five biomarkers would mitigate this issue and improve diagnostic efficacy and accuracy. Biomarkers were combined using a statistical tool called binary logistic regression to generate a new value, “factor R,” which represents the predicting possibility, and was used to avoid the “yes” or “no” issues of single cutoff values. The combination function for differentiating the early-onset subgroup was defined as:


$$\begin{array}{l} R=\;\frac{e^{logit\;\left(g\right)}}{1+e^{logit\;\left(g\right)}} \end{array}$$

i. For AD/non-AD:
*Logit* (*g*) = *Logit* (*g*1) = 0.1950.01 × *Aβ*42 + 0.017 × *t*−*tau* + 0.058 × *p*−*tau* −5.498 × *t*−*tau*/*Aβ*42
ii. For AD/control:
*Logit* (*g*) = *Logit* (*g*2) = 20.130.095 × *Aβ*420.037 × *t*−*tau* + 1.287 × *p*−*tau* +38.52 × *t*−*tau*/*Aβ*42411.05 × *p*−*tau*/*Aβ*42

R infers predicting value based on logistic regression. Variables include *A*β*42, t-tau, p-tau, t-tau/A*β*42, p-tau/A*β*42*. R was handled as a new biomarker for every individual, and was used, along with the original biomarkers, to estimate the diagnostic accuracy and corresponding cutoff values, in Section 3.3.

### 3.3. ROC curves and cutoff values

**Figure 3:**
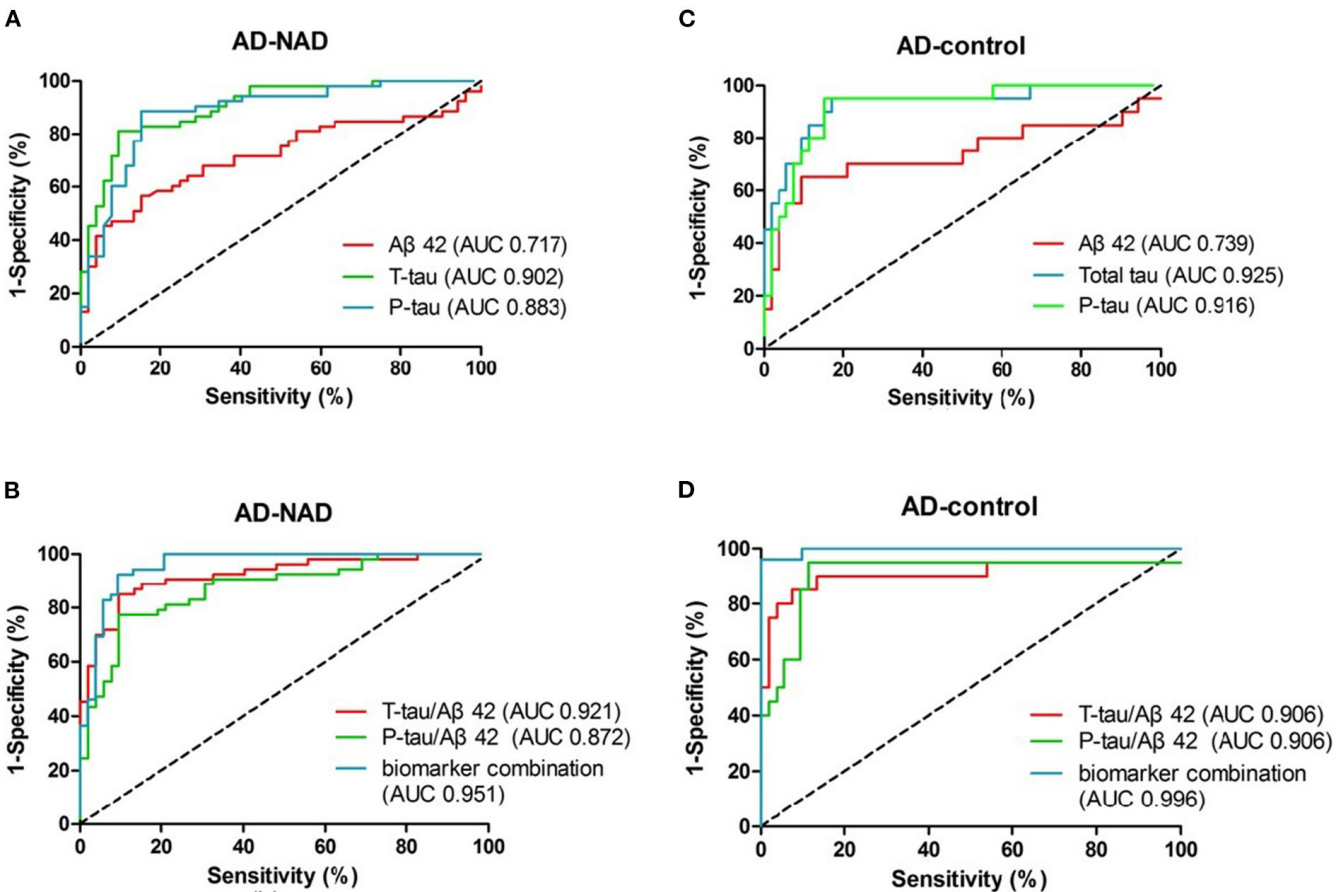
ROC curves of CSF biomarkers in the early-onset group. **(A, C)** depict curves of the original biomarkers. **(B, D)** Display the curves of the calculated ratios and the generated predicting factor, respectively. Diagnostic accuracy, the area under the curve (AUC), is labeled in the legend.

To compare and contrast the results, ROC curves for the five biomarkers and factor R are drawn together in Figure 3. The diagnostic efficacy, estimated as AUC (25), is labeled in the legend in the lower right quadrant. AUCs and their corresponding 95% CIs are described in Table 2. Their corresponding CVs are found in Table 3. To meet the need of optimal clinical use, the CVs reported have highest YI, which means the maximum sensitivity plus specificity. As predicted, we found that the diagnostic efficacy of factor R was higher than the diagnostic efficacies of each of the biomarkers alone [AUC AD vs. non-AD: 0.951, 95% CI (0.911, 0.991); AUC AD vs. control: 0.996, 95% CI (0.988, 1.00)] (Table 2). The second best performer for differentially diagnosing dementia was the t-tau/Aβ42 ratio [AUC AD vs. non-AD: 0.921, 95% CI (0.869, 0.973)], and for distinguishing AD from controls, t-tau [AUC AD vs. control: 0.925, 95% CI (0.852, 0.998)]. According to current guidelines, lumbar puncture and CSF analyses are indicated only for complicated dementia cases; in these cases, based on their maximum YI index, we recommend the following cutoff values for the five measured biomarkers: Aβ42 < 570.9 pg/mL, t-tau > 241.6 pg/mL, p-tau > 56.49 pg/mL, t-tau/Aβ42 ratio > 0.529, and p-tau/Aβ ratio > 0.08465 (Table 3). We found that a cutoff value of 0.4117 for factor R had a higher diagnostic accuracy than each of the five biomarkers alone, with a sensitivity of 86.5 and 100%, and a specificity of 92.4 and 96.2%, for AD vs. non-AD and AD vs. controls, respectively (Table 3).

**Table 2:** AUCs and corresponding 95% CIs.

	**AD vs. NAD**		**AD vs. control**	
	**AUC**	**95% CI**	**AUC**	**95% CI**
Aβ42	0.717	[0.616, 0.818]	0.739	[0.582, 0.896]
T-Tau	0.902	[0.845, 0.960]	0.925	[0.852, 0.998]
^181^P-Tau	0.883	[0.816, 0.949]	0.916	[0.846, 0.987]
T-tau/Aβ42	0.921	[0.869, 0.973]	0.906	[0.799, 1.013]
^181^P-tau/Aβ42	0.872	[0.804, 0.940]	0.906	[0.806, 1.006]
*R*	0.951	[0.911, 0.991]	0.996	[0.988, 1.00]

AD, Alzheimer's disease group; NAD, non-Alzheimer's disease group; R, predicting factor from logistic regression; AUC, area under curve; CI, confidence interval.

**Table 3:** Cutoff values and corresponding sensitivity/specificity.

	**AD vs. NAD**		**AD vs. control**	
	**CV**	**SE%, SP%**	**CV**	**SE%, SP%**
Aβ42 (pg/mL)	< 570.9	56.6, 84.6	< 620.8	65.0, 90.4
T-Tau (pg/mL)	>241.6	81.1, 90.4	>286.8	82.7, 95.0
^181^P-Tau (pg/mL)	>56.49	88.7, 84.6	>55.32	84.6, 95.0
T-Tau/Aβ42	>0.5290	84.9, 90.4	>0.4140	92.3, 85.0
^181^P-tau/ Aβ42	>0.08465	77.4, 90.4	>0.09536	88.5, 95.0
*R*	>0.4117	86.5, 92.4	>0.2464	100, 96.2

CV, cutoff value; SE%, SP%, sensitivity%, specificity%; AD, Alzheimer's disease group; NAD, non-Alzheimer's disease group; R, predicting factor from logistic regression.

## 4. Discussion

Aβ42, both in AD–non-AD differentiation and AD–control differentiation, had relatively low AUC in our study. This low distinguishing power may be due to the wide range of Aβ42 levels (Figure 2) that related to unstability of molecular adhesion. Nevertheless, the other five items were found to have higher diagnostic efficacy than Aβ42 and show promise for use in the clinic. We found that total tau and phosphorylated tau levels distinguished the AD group from the control group best, whereas the best biomarker to distinguish the AD group from the non-AD group was the t-tau/Aβ42 ratio; decreased Aβ42 with increased tau seems specific for AD dementia in degenerative disorders. The predicting factor R, constructed by combining biomarkers, helped to improve the best-individual AUC to 0.951 and 0.996 for AD-non-AD and AD-control groups, respectively. This pattern of data processing may provide a new tool for risk evaluation, with improved prediction capability as more risk factors are revealed.

### 4.1. Demographic and biomarker differences

Early-onset AD has a different pathological distribution than late-onset AD, which makes it confusing in clinical practice (10, 12). CSF biomarkers were found to reflect Aβ and tau pathophysiology consistently among different clinical subtypes. According to the outcomes of our analyses, CSF biomarkers could help in the differential diagnosis of early-onset types of dementia. We demonstrated that the original CSF biomarker levels of Aβ42, t-tau, and p-tau differed significantly between AD and the other two groups tested. Age and MMSE scores did not contribute to the change in CSF biomarker levels (see Section 3.1), which makes it more likely that changes in biomarker levels are disease-specific rather than aging or cognitive status-related at the time, which differs from previous results (26). In some other studies, t-tau was proposed to reflect the neurodegenerative or cognitive stage, and could be used to predict disease process in AD (1, 2). While still controversial, another study reported a similar conclusion to ours (27). This variation may be due to heterogeneity in how tau protein levels change during disease progression. Our future work will investigate the correlation between biomarker protein levels and individual characteristics, such as age, sex, and cognitive function status, in a larger sample population.

### 4.2. Diagnostic accuracy of biomarkers and biomarker combination

Aβ42 reduction is regarded as the earliest criteria in CSF diagnostic profiles (2, 4, 24). However, in our study as well as others, Aβ42 was found to have low diagnostic accuracy. Under our defined CV condition, Aβ42 provided a sensitivity of only 0.55–0.65, but a specificity of 0.84–0.91, meaning it can reliably exclude non-AD patients with normal Aβ42 levels, but fails to detect all patients with AD. This phenomenon may be due to multiple cleavage points in the amyloid protein precursor, which produces different beta-amyloid proteins with various biological functions (28). Another potential cause of Aβ42 diversity is systemic or measurement bias. Measurements of CSF biomarker levels are sensitive to changes in the environment. To reduce the influence of internal and external laboratory differences, we complied with a proposed standard (9). Moreover, we retested all samples in this study and demonstrated that there was no significant bias in measurement either due to analytic performance or long-term storage. Whether the low diagnostic efficacy of Aβ42 is specific to early-onset AD is still unknown.

We found that tau protein, which reflects the destruction of neurons, was more reliable for AD diagnosis, with an AUC around 0.9. We speculate that this may be due to our exclusion of some definite non-AD dementia samples with extremely high t-tau levels, like Creutzfeldt-Jakob disease. For example, some patients with prion disease had t-tau levels over 1,000 pg/mL. Unlike degenerative disorders, these excluded diseases manifest with rapid cognitive decline and can be screened using many other well-developed clinical tests. The tau protein levels in patients with these diseases approaches the average levels seen in AD groups in some other studies (20, 29). This was the reason we revised the exclusion criteria to exclude these diseases. In some basic studies of the etiology and pathology of AD, p-tau correlated well with the neurofibrillary tangles in involved cerebral domains. Hyperphosphorylation of tau protein was once believed to drive tangle formation (30). Very little evidence from our work supports this, as p-tau was not superior in diagnostic accuracy (AUC and YI) compared with t-tau. One explanation is that p-tau in neurofibrillary tangles does not escape into CSF, which means that p-tau in CSF does not reflect the substantial p-tau burden in the brain. New positron-emission tomography/CT tracers for p-tau could be used to test this hypothesis in the future (31).

Biomarker combination, like some other established predicting functions, is often more accurate than using the original biomarkers (21, 22, 32). In our study we found that the t-tau/Aβ42 ratio, with an AUC of 0.92, is excellent at differentially diagnosing dementia. This can be explained using mathematics theory which says that the ratio expands when the numerator increases and the denominator decreases. The difference between the ratios in each group therefore broadens giving a better definition of an optimal cutoff value. However, this ratio was not more useful at screening AD patients from control patients, compared with total tau alone. We speculate that elevated tau plus decreased Aβ42 is somehow specific to AD or other degenerative disorders, rather than non-degenerative neurological diseases. Changes to Aβ42 and tau levels vary differently in those non-degenerative diseases, which makes the ratio less effective in this field. Unexpectedly, we found that combining biomarkers and their ratios further into a logistic regression generated factor R increased the AUCs to a range over 0.95. This higher diagnostic accuracy means that this predicting function can be used as an alternative to the original biomarker CVs and diagnostic decision trees (20). We believe that factor R could help us confidently predict the diagnostic possibility of a patient with borderline biomarker levels.

### 4.3. Limitations

Our study has several limitations. First, our sample sizes for the non-AD and the non-cognitive disturbance group were small. The non-AD group was composed of various diseases, with limited numbers of each type, as described in Section 2.2. Intrinsic variances of biomarker levels from different dementias distort the biomarker distributions from a normal Gaussian distribution, which introduces bias into the analysis of non-AD dementia. Some extreme conditions, like Creutzfeldt-Jakob disease and brain metastasis, that show greatly elevated t-tau levels (over 1,000 pg/mL), were excluded from the non-AD group of our analysis to reduce this bias. Second, the diagnoses we made were all clinically based, and lacked conclusive postmortem autopsy evidence, which may have introduced diagnostic errors. Third, patients visiting our center are more severely cognitively impaired. Thus, the cognitive decline in our AD group was more severe than the other groups, and even than that in some other studies (20, 29). The main purpose of our study was to detect early-stage AD; thus, patients with relatively mild cognitive impairment will need to be studied next.

## 5. Conclusion

In conclusion, the biomarkers we investigated, Aβ42, total-tau, p-tau, and the t-tau/Aβ42, p-tau/Aβ42 ratios, can be used to differentiate AD dementia from other types of degenerated dementia patients, in early-onset subgroups. The functions we constructed raised the prediction accuracy of the biomarkers to even higher levels, highlighting the importance of combining biomarkers. Our cutoff values, based on a Chinese population from PUMCH dementia cohort, were similar to previous reports (22, 29, 32). Clinical practice with biomarker cutoff values would improve early diagnosis. As far as we know, we are the first to report on these biomarkers in the exclusive Chinese population. Thus, our work not only expands on a novel tool for clinical AD diagnosis in our center, and it may become part of the reference data standard for CSF biomarker diagnosis of AD worldwide. Our future work will focus on increasing recruitment from our clinics to create a more representative dataset, thereby reducing bias and error.

## Data availability statement

The raw data supporting the conclusions of this article will be made available by the authors, without undue reservation.

## Ethics statement

The studies involving human participants were reviewed and approved by Institutional Review Board of the Peking Union Medical College Hospital (No. ZS2009). The patients/participants provided their written informed consent to participate in this study.

## Author contributions

DL performed the data analyses, samples collection, and wrote the manuscript. CM helped perform the analysis with constructive discussions. LS performed samples storage and ELISA analyses. XH, JL, CL, and LD helped to perform cognitive and systematic assessment. QX possessed the sample storage and basic science laboratory. JG contributing to the conception, construction, and fund of this whole study. All authors contributed to the article and approved the submitted version.
